# Eriodictyol Attenuates H_2_O_2_-Induced Oxidative Damage in Human Dermal Fibroblasts through Enhanced Capacity of Antioxidant Machinery

**DOI:** 10.3390/nu14122553

**Published:** 2022-06-20

**Authors:** Visarut Buranasudja, Chawanphat Muangnoi, Kittipong Sanookpan, Hasseri Halim, Boonchoo Sritularak, Pornchai Rojsitthisak

**Affiliations:** 1 Department of Pharmacology and Physiology, Faculty of Pharmaceutical Sciences, Chulalongkorn University, Bangkok 10330, Thailand; visarut.b@pharm.chula.ac.th (V.B.); sanookpan.k@gmail.com (K.S.); 2 Center of Excellence in Natural Products for Ageing and Chronic Diseases, Chulalongkorn University, Bangkok 10330, Thailand; boonchoo.sr@chula.ac.th; 3 Institute of Nutrition, Mahidol University, Nakhon Pathom 73170, Thailand; chawanphat.mua@mahidol.ac.th; 4 Faculty of Pharmacy, Universiti Teknologi MARA (UiTM) Cawangan Selangor, Kampus Puncak Alam, Bandar Puncak Alam 42300, Selangor, Malaysia; hasseri2945@uitm.edu.my; 5 Integrative Pharmacogenomics Institute (iPROMISE), Universiti Teknologi MARA, Bandar Puncak Alam 42300, Selangor, Malaysia; 6 Department of Pharmacognosy and Pharmaceutical Botany, Faculty of Pharmaceutical Sciences, Chulalongkorn University, Bangkok 10330, Thailand; 7 Department of Food and Pharmaceutical Chemistry, Faculty of Pharmaceutical Sciences, Chulalongkorn University, Bangkok 10330, Thailand

**Keywords:** fibroblasts, oxidative stress, eriodictyol, hydrogen peroxide, skin aging, antioxidants

## Abstract

Oxidative stress in dermal fibroblasts is strongly correlated with the aging process of the skin. The application of natural compounds that can increase the ability of dermal fibroblasts to counteract oxidative stress is a promising approach to promote skin health and beauty. Eriodictyol is a flavonoid that exerts several pharmacological actions through its antioxidant properties. However, its protective effects on dermal fibroblasts have not yet been investigated. In this study, we investigated whether eriodictyol protects human dermal fibroblasts (BJ fibroblasts) from the harmful effects of hydrogen peroxide (H_2_O_2_). Eriodictyol pretreatment significantly prevented necrotic cell death caused by H_2_O_2_ exposure. In addition, the level of 2′,7′-dichloro-dihydro-fluorescein oxidation was decreased, and that of glutathione was maintained, indicating that the beneficial effects of eriodictyol against H_2_O_2_ were closely associated with oxidative-stress attenuation. Eriodictyol mediates its antioxidant effects on dermal fibroblasts against H_2_O_2_ through (i) the direct neutralization of reactive oxygen species; (ii) the enhancement of the activities of H_2_O_2_-detoxifying enzymes, including catalase and glutathione peroxidase; and (iii) the induction of the expressions of catalase and glutathione peroxidase 1 via the activation of the Nrf2 signaling system. These results support the potential application of eriodictyol as an ingredient in skincare products for cosmeceutical and pharmaceutical purposes.

## 1. Introduction

In humans, the skin is the organ with the largest surface area and serves as the first protective barrier between internal organs and the external environment. Similar to other tissues, the skin undergoes progressive alterations over time. In contrast to other organs, the skin, as the outermost boundary, is constantly exposed to diverse kinds of environmental stressors, such as ultraviolet radiation, tobacco smoke, pollution, and toxic chemicals. Exposure to these external insults can lead to the formation and accumulation of reactive oxygen species (ROS), with a resultant increase in oxidative stress, eventually accelerating the process of skin aging. Therefore, increased ROS generation and oxidative stress are believed to be important driving forces for skin aging [1,2,3].

The skin is organized into three primary layers: epidermis, dermis, and hypodermis. The dermis is a region of dense, irregular connective tissues that confer strength, elasticity, and extensibility to the skin. Fibroblasts are the predominant cell type in the dermis. An essential function of dermal fibroblasts is to produce an extracellular matrix, including collagenous and elastin fibers. Collagenous fibers are necessary for the tensile strength and mechanical properties of the skin, whereas elastin fibers are responsible for the overall elasticity of the skin [4]. Exposure to environmental and xenobiotic insults can disrupt the function of dermal fibroblasts through oxidative stress. Consequently, fibroblast dysfunction impairs the structural and mechanical integrity of the skin, eventually resulting in skin aging [5,6,7,8,9,10,11]. Therefore, using compounds that can promote the ability of dermal fibroblasts to counteract oxidative imbalance would be a promising strategy for inhibiting age-related skin problems.

The progression of skin aging is strongly correlated with oxidative-stress generation in skin cells [12]. Therefore, there has been considerable interest in cosmeceutical research to identify potential candidates with antioxidant properties to decrease the formation of detrimental ROS and prevent the acceleration of skin aging. Several antioxidants from plants have been widely used as bioactive agents in cosmeceuticals to strengthen and protect skin cells against environmental stimuli [13,14]. Flavonoids are among the largest and most widespread secondary metabolites in plants, with significant antioxidant activity [15]. The proposed protective effects underlying their antioxidant activity are (1) the attenuation of ROS generation by either the inhibition of endogenous enzyme activity or the chelation of redox metals involved in ROS generation [16,17,18], (2) the direct scavenging of ROS [16], and (3) the increased expression or activity of cellular antioxidant machinery [15,18]. Because of their potent antioxidant activity, flavonoids are an attractive class of phytochemicals that can be used against age-related skin disorders.

Eriodictyol is a flavonoid that exerts a wide range of pharmacological actions via its antioxidant property. Previously, eriodictyol was reported to prevent oxidative-stress-induced cell death in an in vitro model of cardiovascular disorder, and eriodictyol pretreatment significantly inhibited oxidative DNA damage in H_2_O_2_-treated endothelial cells [19]. Consistent with the findings in the cell culture model, in another study, eriodictyol attenuated oxidative injury in an in vivo model of acute lung injury using lipopolysaccharide (LPS) as a stressor and significantly inhibited pulmonary injury and lung inflammation in LPS-treated mice by alleviating excessive oxidative damage [20]. The beneficial effects of eriodictyol were also observed in a preclinical model of age-related macular degeneration. Eriodictyol significantly inhibited hydrogen peroxide (H_2_O_2_)- and *t*-butyl hydroperoxide-induced cytotoxicity by inhibiting intracellular oxidative-stress induction in human retinal pigment epithelial cells [21,22]. Eriodictyol also demonstrated protective action against streptozotocin-induced diabetic retinopathy in vivo and significantly attenuated retinal inflammation in diabetic rats by inhibiting oxidative damage [23]. Collectively, these findings show that eriodictyol has potential therapeutic application in oxidative-stress-associated problems, including skin aging.

In this study, we aimed to evaluate the beneficial activities of eriodictyol isolated from *Dendrobium ellipsophyllum* against oxidative damage in human dermal fibroblasts. Our findings support the potential utility of eriodictyol for pharmaceutical and cosmeceutical purposes to maintain skin health and beauty.

## 2. Materials and Methods

### 2.1. Chemicals and Reagents

H_2_O_2_, 3-(4,5-dimethylthiazol-2-yl)-2,5-diphenyltetrazolium bromide (MTT), 2′,7′-dichloro-dihydro-fluorescein diacetate (DCFH-DA), 2,2-diphenyl-1-picrylhydrazyl (DPPH), Trolox, dimethyl sulfoxide (DMSO), and Triton x-100 were purchased from Millipore Sigma (Burlington, MA, USA). In addition, cell culture media and supplements (minimum essential medium (MEM), pyruvate, penicillin/streptomycin, and fetal bovine serum (FBS)) were purchased from Gibco (Waltham, MA, USA).

### 2.2. Eriodictyol Preparation

Eriodictyol was isolated from the whole plant of *D. ellipsophyllum* as previously described [24]. Briefly, we macerated 4.8 kg of dried and powdered whole *D. ellipsophyllum* plant with methyl alcohol (MeOH), removed the solvent, and obtained 400 g of MeOH extract. Next, 200 g of MeOH extract was subjected to vacuum liquid chromatography over silica gel (hexane-EtOAc, gradient) to obtain five fractions (A–E). Subsequently, 63 g of fraction D was further fractionated by column chromatography over silica gel (hexane-EtOAc, gradient) to obtain seven fractions (D1–D7), and 5.4 g of fraction D5 was subjected to medium-pressure liquid chromatography over silica gel (hexane-EtOAc, gradient) and then further purified by column chromatography over silica gel (hexane-EtOAc, gradient) to obtain 364 mg of eriodictyol. The chemical structure of the obtained eriodictyol was verified using nuclear magnetic resonance (NMR) spectroscopy.

(2*S*)-Eriodictyol: colorless needles; C_15_H_12_O_6_; ESI-MS *m/z* 289 [M+H]^+^; ${\left[\alpha \right]}_{D}^{20}$ −18.7 (*c* = 0.2, MeOH); ^1^H-NMR (500 MHz, acetone-*d*_6_) δ: 2.72 (1H, dd, *J* = 17.0, 3.0 Hz, H-3*_cis_*), 3.11 (1H, dd, *J* = 17.0, 12.5 Hz, H-3*_trans_*), 5.37 (1H, dd, *J* = 12.5, 3.0 Hz, H-2), 5.93 (1H, d, *J* = 2.0 Hz, H-6), 5.95 (1H, d, *J* = 2.0 Hz, H-8), 6.86 (2H, d, *J* = 1.5 Hz, H-5′, H-6′), 7.02 (1H, d, *J* = 1.5 Hz, H-2′), 12.16 (1H, s, HO-5); ^13^C-NMR (12_5_ MHz, acetone-*d*_6_) δ: 43.4 (C-3), 79.9 (C-2), 95.8 (C-8), 96.7 (C-6), 103.1 (C-10), 114.6 (C-2′), 115.9 (C-5′), 119.2 (C-6′), 131.5 (C-1′), 145.9 (C-3′), 146.3 (C-4′), 164.2 (C-5), 165.2 (C-9), 167.2 (C-7), 197.1 (C-4). The purity of eriodictyol was measured by HPLC. Eriodictyol with more than 98% purity was used in this study.

### 2.3. Free Radical Scavenging Assay

DPPH is a stable violet-colored free radical with strong absorption at 517 nm and is used to determine the radical scavenging activity (RSA) of compounds. A sample possessing antioxidant activity can donate an electron or hydrogen atom to DPPH (purple) and generate a reduced form of DPPH (pale yellow). The rate of color change from purple to yellow is directly proportional to the RSA of the sample.

In this study, we added serial dilutions of eriodictyol in methanol (0–600 µM; 20 µL) to 96-well plates, mixed the samples with DPPH in methanol (150 µM; 180 µL), and incubated the samples in the dark at room temperature. After 30 min, the absorbance was measured at a wavelength of 517 nm using a CLARIOStar microplate reader (BMG Labtech, Germany). Trolox was used as a positive control. The %RSA was calculated as follows:

%RSA = ((*A*_blank_ − *A*_sample_)/*A*_blank_) × 100

where *A*_blank_ and *A*_sample_ are the absorbances of the blank and sample, respectively. We estimated the concentration of compounds that resulted in a 50% reduction in DPPH absorbance from a concentration–response curve using GraphPad Prism version 9.2.0 (GraphPad Software, San Diego, CA, USA).

### 2.4. Cell Culture

Normal human foreskin fibroblast cell line BJ (ATCC No. CRL-2522) was obtained from the American Type Culture Collection (ATCC, Manassas, VA, USA). BJ cells were cultured in MEM supplemented with 1 mM pyruvate, 10% FBS, 100 U/mL penicillin, and 100 µg/mL streptomycin at 37 °C in a humidified atmosphere with 95% air/5% CO_2_.

### 2.5. Eriodictyol Treatment of H_2_O_2_-Exposed BJ Cells

We used H_2_O_2_ as an oxidative-stress inducer. For each experiment, we freshly prepared a working solution of H_2_O_2_ in serum-free MEM. To investigate H_2_O_2_-induced cytotoxicity, BJ cells were exposed to 125–1000 µM H_2_O_2_ for 1 h. For the control, we replaced the medium with serum-free MEM similar to that for BJ cells exposed to H_2_O_2_.

To determine the protective activities of eriodictyol against H_2_O_2_-induced cytotoxicity, we pre-incubated BJ cells with eriodictyol (final concentration, 10–40 µM; 0.5% DMSO) for 24 h prior to H_2_O_2_ exposure. The control group was incubated with an equivalent amount of 0.5% DMSO, which is considered a nontoxic concentration for BJ cells.

### 2.6. Cell Viability Assay

BJ cells were seeded into 96-well plates at a density of 20,000 cells/well in 200 µL of culture medium and cultured for 24 h before exposure to the experimental conditions. After the indicated treatments, the medium was aspirated, and the cells were washed twice with phosphate-buffered saline (PBS; pH 7.4). Cell viability was evaluated with the MTT assay. Briefly, we incubated the washed cells in 200 µL of MTT solution (1 mg/mL in serum-free medium) in the dark for 3 h, removed the MTT solution, and solubilized the reduced formazan crystals by adding 200 µL of DMSO into each well. The absorbance of the formazan solution was measured at a wavelength of 570 nm using a CLARIOStar microplate reader.

### 2.7. Apoptosis and Necrosis Detection

The detection of cell death was assessed by dual fluorescent labeling with Hoechst 33342 and propidium iodide (PI). Hoechst 33342 is a nuclear counterstain with a cell-permeable property for the visualization of apoptotic characters, e.g., chromatin condensation and DNA fragmentation. PI is a membrane-impermeable dye that can be intercalated to the DNA of cells with a loss of membrane integrity (necrotic cells) [25]. Hence, Hoechst 33342 and PI are nuclear counterstains widely used to distinguish the mode of cell death.

Following treatments, cells were co-stained with 10 µM Hoechst 33342 and 5 µM PI (Sigma Chemical, St. Louis, MO, USA) for 30 min in the dark at 37 °C. Cell morphological changes were captured and visualized using a fluorescence microscope (Nikon ECLIPSE Ts2; Tokyo, Japan).

### 2.8. Determination of the Cellular Redox Status

Oxidant-sensing probe DCFH-DA was used to evaluate the redox status of BJ cells following treatment. The DCFH-DA probe is cell-permeable and subjected to deacetylation within the cell by esterases to generate nonfluorescent DCFH, which is retained in the cytosol. In the presence of ROS and other oxidants, DCFH is oxidized to fluorochrome 2′,7′-dichlorofluorescein. Therefore, this probe has been used as an indicator of oxidative stress in biological systems [26,27].

The cell culture condition for determining the cellular redox status was similar to that of the cell viability assay. After eriodictyol treatment, BJ cells were washed twice with PBS and incubated with 5 µM DCFH-DA in serum-free medium for 30 min. Subsequently, the cells were washed twice with PBS prior to H_2_O_2_ exposure. After treatment for 1 h, the medium was aspirated and replaced with serum-free medium. Finally, fluorescence was determined at an excitation wavelength of 485 nm and an emission wavelength of 530 nm using a CLARIOStar microplate reader.

### 2.9. Measurement of Glutathione (GSH) Levels

BJ cells were seeded into 6-well plates at a density of 1 × 10^6^ cells/2 mL of MEM and cultured for 24 h. After the indicated treatments, the cells were washed with cold PBS, harvested using a cell scraper, suspended in 250 µL of PBS containing 0.5% Triton X-100, and sonicated in an ice-cold water bath for 15 min for cell lysis. Next, the cell lysate was centrifuged at 10,000× *g* for 5 min at 4 °C, and the supernatant was collected to evaluate the GSH level using a GSH assay kit (Cayman Chemical, Ann Arbor, MI, USA) according to the manufacturer’s instructions without modifications.

Briefly, the assay reagent was freshly prepared prior to the measurement by mixing the following reagents: cofactor mixture solution (NADP^+^ and glucose-6-phosphate; 0.45 mL), enzyme mixture solution (glutathione reductase and glucose-6-phosphate dehydrogenase; 2.1 mL), 5,5′-dithio-bis-(2-nitrobenzoic acid) (DTNB; 0.45 mL), assay buffer (11.25 mL), and water (2.3 mL). To initiate the redox-cycling reaction, cell lysate (50 μL) was mixed with the assay reagent (150 μL) and incubated in the dark for 25 min. The absorbance at 405 nm was measured with a CLARIOStar microplate reader. Standard curves for each measurement were generated by GSSG stock solutions (0–8 μM final concentration). Under the experimental conditions, 1 mole of GSSG was reduced to generate 2-mole equivalents of GSH. The intracellular GSH concentrations of samples were then calculated from the corresponding standard curve. The GSH levels were normalized with total protein content.

### 2.10. Evaluation of Antioxidant Enzyme Activity

The cell culture conditions, treatments, and cell lysate preparations for the evaluation of antioxidant enzyme activity were similar to those for measuring GSH levels. Catalase (CAT) and glutathione peroxidase (GPx) activities in the cell lysate supernatants were determined with 96-well-microplate format using the catalase assay kit (Cayman Chemical) and the glutathione peroxidase assay kit (Cayman Chemical), respectively.

For the catalase assay, cell lysate (20 μL) was gently mixed with methanol (30 μL) and assay buffer (100 μL) in each experimental well. To initiate the peroxidatic reaction, H_2_O_2_ solution (20 μL; 35 mM) was introduced into a lysate solution. The major end product of this reaction is formaldehyde. Hence, the rate of formaldehyde production from the peroxidatic reaction can be used to estimate catalase activity. After 20 min, potassium hydroxide (30 μL; 10 M) was added to terminate the reaction. The analyzed sample was subsequently incubated with 4-amino-3-hydrazino-5-mercapto-1,2,4-triazole (purpaid; 30 μL) for 10 min. Purpaid is used as a chromogen to specifically interact with generated formaldehyde from peroxidatic reaction. Potassium periodate (10 μL; 0.5 M) was then added into each well to complete the oxidation reaction and generate a purple-colored product. After 5 min of incubation, the alteration in color (from colorless to purple) was observed at a wavelength of 540 nm using a CLARIOStar microplate reader. Standard curves with serial dilutions of formaldehyde solution (0–85 μM) were used in each experiment to estimate the amount of generated formaldehyde in the sample. The activity of catalase in the sample was further calculated as units per milligram of protein. One unit of catalase is defined as the amount of catalase that generates 1.0 nmol formaldehyde under the experimental condition specified above.

For the GPx assay, cell lysate (20 μL) was mixed with the co-substrate mixture (glutathione reductase and GSH; 50 μL), NADPH (50 μL) and assay buffer (50 μL) in each experimental well. A cumene hydroperoxide solution (20 μL) was added to the lysate solution to initiate redox reactions. After adding cumene hydroperoxide, the absorbance at 340 (A_340_) was monitored every 1 min for 10 min with a CLARIOStar microplate reader. The GPx activity in lysate samples was then calculated from the rate of A_340_ decrease of samples. The GPx activity was expressed as units per milligram of protein. One unit of GPx is defined as the amount of GPx that produces 1.0 nmol NADP^+^ from NADPH in the experimental condition specified above.

### 2.11. Western Blot Analysis

Following treatments, cells were harvested and lysed with RIPA buffer containing a Protease/Phosphatase inhibitor cocktail (Cat. No. 5872; Bio-Rad Laboratories, Hercules, CA, USA). The protein concentrations were estimated using a Pierce BCA protein assay kit (Cat. No. 23225; Thermo Fisher Scientific, Waltham, MA, USA). Equal amounts of protein lysates were separated by electrophoresis on sodium dodecyl sulfate (SDS)-polyacrylamide gels and transferred into a PVDF membrane (Bio-Rad Laboratories, Hercules, CA, USA). The membrane was blocked with 5% non-fat dry milk in TBS-T for 1 h at 25 °C and probed with the following primary antibodies at 4 °C overnight: anti-Nrf2 (1:1000; Cat. No. ab137550; Abcam, Cambridge, UK), anti-glutathione peroxidase 1 (1:1000; Cat. No. ab22604; Abcam, Cambridge, UK), anti-catalase (1:1000; Cat. No. 12980; Cell Signaling, Danvers, MA, USA), anti-HO-1 (1:1000; Cat. No. 43966; Cell Signaling, Danvers, MA, USA), anti-Keap1 (1:1000; Cat. No. 8047; Cell Signaling, Danvers, MA, USA). Anti-GAPDH (1:1000; Cat. No. 5174; Cell Signaling, Danvers, MA, USA) was used as a loading control. The membrane was then washed 3 times with TBS-T and incubated with anti-rabbit horseradish peroxidase-linked secondary antibodies (1:2000; Cat. No. 7074; Cell Signaling, Danvers, MA, USA) at 25 °C for 1 h. Subsequently, the signal was developed using Immobilon Western chemiluminescent HRP substrate (Cat. No. P90719; Millipore Sigma, Burlington, MA, USA) and visualized with an ImageQuantTM LAS 4000 biomolecular imager (GE Healthcare, Chicago, IL, USA). The protein band intensity was quantified using ImageJ software (version 1.53r; U.S. National Institutes of Health, Bethesda, MD, USA).

### 2.12. Statistical Analysis

GraphPad Prism version 9.3.1 (GraphPad Software Inc., San Diego, CA, USA) was used for the statistical analyses. The unpaired *t*-test and one-way analysis of variance with Tukey’s post hoc test were performed to calculate statistical differences. All means and standard errors of the mean (SEMs) were estimated from at least three independent experiments. Data were presented as the mean ± SEM.

## 3. Results

### 3.1. H_2_O_2_ Exposure Caused Cytotoxic Effects in Dermal Fibroblasts

**Figure 1:**
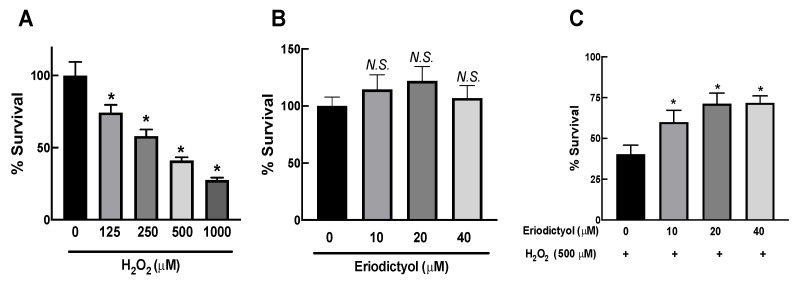
Eriodictyol inhibited H_2_O_2_-induced cytotoxicity in dermal fibroblasts. (**A**) H_2_O_2_ exposure (125–1000 µM; 1 h) resulted in a concentration-dependent decrease in the viability of BJ cells. (**B**) Eriodictyol treatment (10–40 µM; 24 h) did not result in cytotoxicity in BJ cells. (**C**) Eriodictyol pretreatment (10–40 µM; 24 h) prevented H_2_O_2_-induced cytotoxicity (500 µM; 1 h) in BJ cells. Cell viability was observed using the MTT assay after the indicated treatments. The survival percentage was calculated relative to untreated controls. DMSO (0.5%) was used as a vehicle control (*n* = 3; each biological replicate consisted of four independent wells; mean ± SEM). * *p* < 0.05 versus vehicle controls (**A**) and *p* < 0.05 versus H_2_O_2_-treated cells (**C**). *N.S.*, not significant.

Skin aging strongly correlates with oxidative stress in dermal fibroblasts [12]. Oxidative damage to dermal fibroblasts can be investigated with the MTT assay using BJ cells as a dermal fibroblast model and H_2_O_2_ as an oxidative-stress inducer [28,29,30,31,32,33]. In this study, the viability of BJ cells gradually reduced in a concentration-dependent fashion after 1 h of H_2_O_2_ exposure (Figure 1A). We selected 500 µM H_2_O_2_ to induce oxidative injury in BJ cells to evaluate the inhibitory activities of eriodictyol.

### 3.2. Eriodictyol Attenuated the Cytotoxicity of H_2_O_2_ in Dermal Fibroblasts

First, we determined the potential cytotoxicity of eriodictyol in dermal fibroblasts. After incubation for 24 h, up to 40 µM eriodictyol did not affect the viability of BJ cells (Figure 1B). Therefore, we selected nontoxic concentrations of eriodictyol ranging from 0 to 40 µM to further investigate the protective activities of eriodictyol against oxidative imbalance. Eriodictyol pretreatment of BJ cells at nontoxic concentrations for 24 h prior to H_2_O_2_ exposure significantly inhibited H_2_O_2_-induced cytotoxicity (Figure 1C), indicating the protective effect of eriodictyol, possibly owing to its antioxidant activity.

### 3.3. Eriodictyol Prevents H_2_O_2_-Induced-Necrotic Cell Death

**Figure 2:**
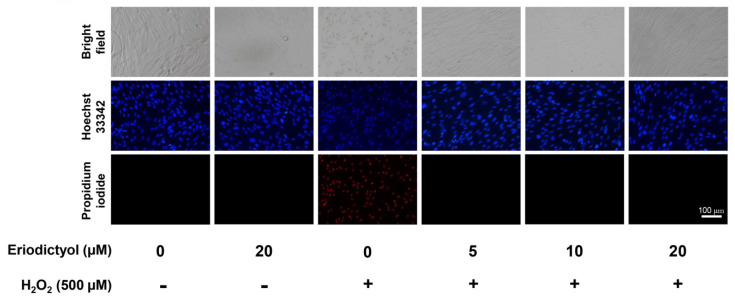
Eriodictyol prevented the induction of necrosis in BJ cells following H_2_O_2_ treatment. BJ cells were pre-treated with eriodictyol (5–20 µM; 24 h) followed by H_2_O_2_ treatment (500 µM; 1h). After 24 h treatment with H_2_O_2_, dermal fibroblasts were co-stained with Hoechst 33342 (blue)/PI (red) to characterize mode of cell death (*n* = 3; magnification = 20×; scale bar = 100 µm).

Following 24 h of H_2_O_2_ treatment, the morphological appearance of dermal fibroblasts altered from spindle-shaped to rounded and swollen cells (Figure 2; bright field). These changes in cellular morphology are the characteristic of necrotic cell death [34,35]. To further identify and characterize the mode of cell death, dermal fibroblasts were double-labeled with Hoechst 33342 and propidium iodide (PI) after treatments. For the H_2_O_2_-treated group, necrotic cells (PI-positive cells) were observed after 24 h of treatment, whereas cells with apoptotic morphology were not found (Figure 2). These data suggest that necrosis is the primary factor for cytotoxicity of H_2_O_2_ in this experimental setting. Notably, apoptotic and necrotic events did not rapidly occur after H_2_O_2_ treatment (Supplementary Figure S1).

Under light microscopy, eriodictyol prevented the changes in the morphological features of necrosis following H_2_O_2_ exposure (Figure 2; bright field), suggesting an anti-necrotic activity of this flavonoid. Furthermore, the fluorescence microscopic images with Hoechst 33342/PI dual-staining demonstrated that supplementation with eriodictyol (5–20 µM) strongly prevented necrosis induction following exposure to H_2_O_2_ (Figure 2; absence of PI-positive cells). Altogether, these results suggest that eriodictyol could inhibit oxidative injury of H_2_O_2_ through the inhibition of necrotic cell death.

### 3.4. Eriodictyol Inhibited H_2_O_2_-Induced Oxidative Stress

**Figure 3:**
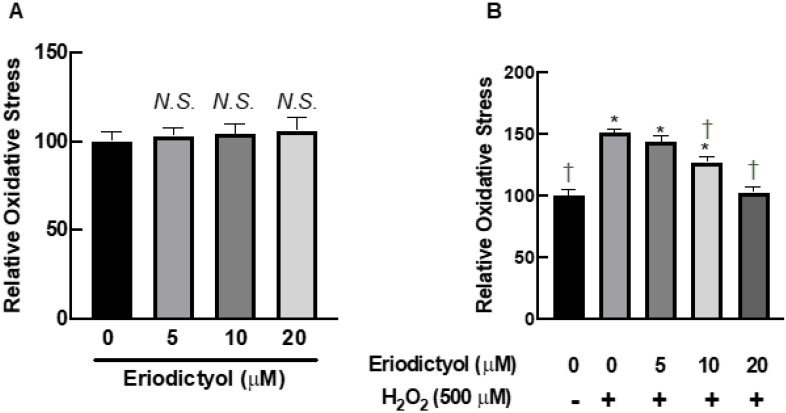
Eriodictyol inhibited oxidative stress in BJ cells following H_2_O_2_ exposure. (**A**) Eriodictyol treatment (5–20 µM; 24 h) did not alter the redox status of BJ cells. (**B**) Eriodictyol pretreatment (10–20 µM; 24 h) inhibited H_2_O_2_-induced oxidative stress (500 µM; 1 h) in a concentration-dependent fashion. Oxidative stress was immediately evaluated using the DCFH-DA assay after the indicated treatments. Relative oxidative stress was calculated compared with that in untreated controls. DMSO (0.5%) was used as a vehicle control (*n* = 3; each biological replicate consisted of three independent wells; mean ± SEM). * *p* < 0.05 versus untreated controls; ^†^ *p* < 0.05 versus H_2_O_2_-treated cells. *N.S.*, not significant.

The protective effects of eriodictyol on cell viability after H_2_O_2_ exposure (Figure 1C and Figure 2) are possibly due to oxidative-stress inhibition. We observed the activity of eriodictyol against oxidative imbalance using DCFH-DA as a probe to monitor the cellular redox status. Eriodictyol treatment for 24 h did not disrupt the redox balance of BJ cells (Figure 3A). However, H_2_O_2_ exposure significantly generated oxidative stress in BJ cells, as shown by an increase of DCFH oxidation by 50% compared with that in the control group (Figure 3B). Eriodictyol pretreatment significantly inhibited H_2_O_2_-induced oxidative stress in a concentration-dependent fashion. These data show that H_2_O_2_-generated oxidative stress could be completely prevented with 20 µM eriodictyol treatment (Figure 3B), indicating that eriodictyol may prevent oxidative damage by inhibiting oxidative stress.

**Figure 4:**
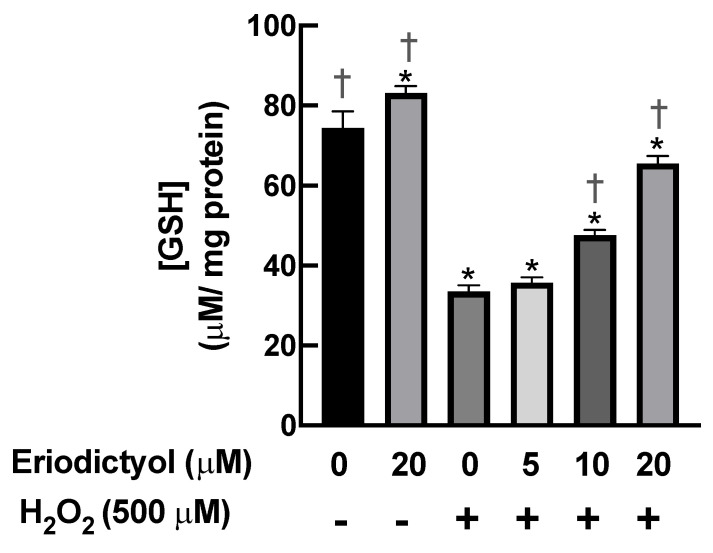
Eriodictyol restored the intracellular levels of reduced glutathione following H_2_O_2_ exposure. H_2_O_2_ (500 µM; 1 h) rapidly decreased the availability of GSH in BJ cells. Eriodictyol pretreatment (10–20 µM; 24 h) maintained the intracellular levels of GSH in a concentration-dependent fashion. DMSO (0.5%) was used as a vehicle control (*n* = 3; each biological replicate consisted of three independent wells; mean ± SEM). * *p* < 0.05 versus vehicle controls; ^†^ *p* < 0.05 versus H_2_O_2_-treated cells.

GSH is an essential marker of the redox environment in biological systems. Cellular redox homeostasis disruption by oxidative stress can reduce GSH levels, resulting in the development of various diseases [36]. To validate the antioxidant activity of eriodictyol against oxidative stress, we determined the GSH levels in BJ cells following H_2_O_2_-generated oxidative stress in the presence and absence of eriodictyol. The intracellular GSH levels were significantly decreased after 1 h of H_2_O_2_ exposure. Pretreatment with eriodictyol significantly prevented the H_2_O_2_-induced decrease in GSH levels in a concentration-dependent manner; specifically, 10 and 20 µM eriodictyol enhanced intracellular GSH levels by 42% and 100%, respectively, following H_2_O_2_ exposure (Figure 4). Collectively, these results of DCFH-DA-based and GSH assays show that eriodictyol could prevent the cytotoxicity of oxidative insults by inhibiting oxidative stress.

### 3.5. Eriodictyol Ameliorated Oxidative Damage by Enhancing Radical Scavenging Capacity

**Figure 5:**
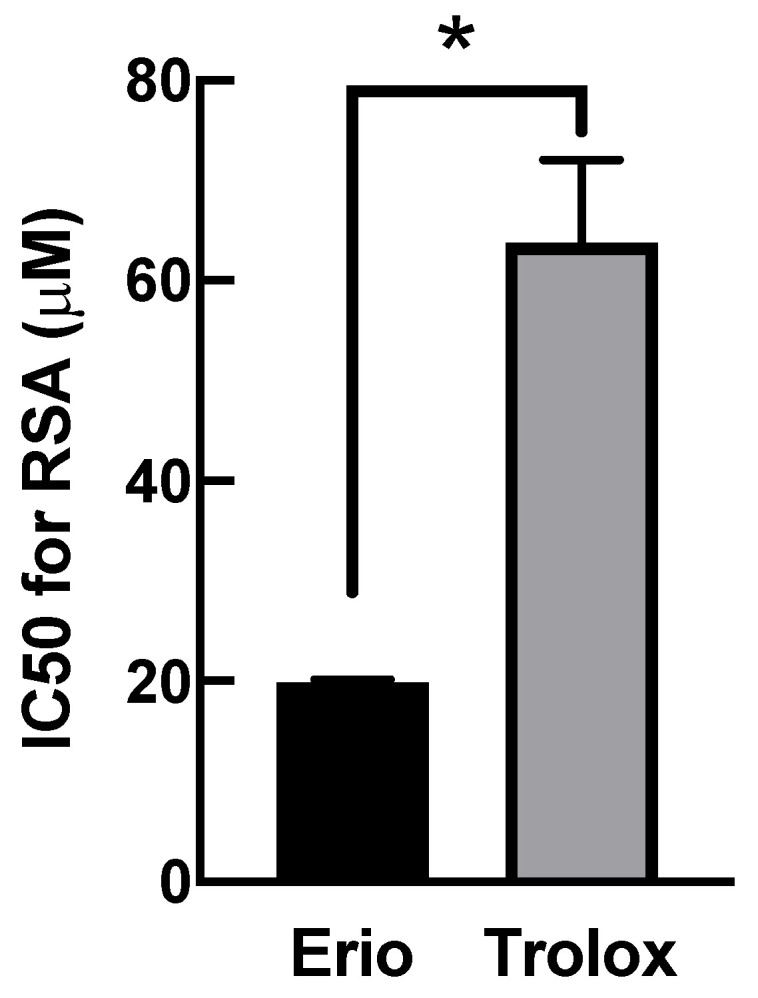
Eriodictyol has potent radical scavenging activity (RSA). The RSA of compounds was evaluated using the DPPH assay. The IC_50_ of eriodictyol required to decrease the radical activity of DPPH was calculated. The IC_50_ for the RSA of eriodictyol was 3.2-fold greater than that of Trolox (eriodictyol, 19.9 ± 0.3 µM versus Trolox, 63.8 ± 8.3 µM; *n* = 3; mean ± SEM, * *p* < 0.05).

The inhibition of oxidative stress by eriodictyol (Figure 1, Figure 2, Figure 3 and Figure 4) could be attributable to three underlying pharmacological activities: (i) eriodictyol directly neutralizes free radicals; (ii) eriodictyol enhances the activity of cellular antioxidant machinery; or (iii) eriodictyol promotes the expression of antioxidant system. To analyze the first beneficial effect, we investigated the direct scavenging capability of eriodictyol using the conventional DPPH assay. The comparative analysis of the half-maximal inhibitory concentration (IC_50_) for DPPH radical inhibition showed that eriodictyol had a 3.2-fold greater ability to neutralize free radicals than that of Trolox, a reference antioxidant (Figure 5). The results of this cell-free-based approach suggest that eriodictyol has strong free radical scavenging ability. Therefore, a potential activity by which eriodictyol protects dermal fibroblasts from the harmful effects of oxidative damage is the direct removal of free radicals.

### 3.6. Eriodictyol Prevented H_2_O_2_-Induced Cell Damage by Enhancing Cellular Antioxidant Enzyme Activity

**Figure 6:**
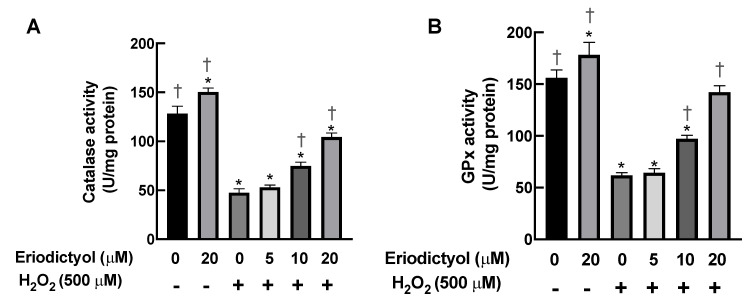
Eriodictyol enhances the activities of CAT and GPx. (**A**,**B**) Eriodictyol pretreatment (10–20 µM; 24 h) promoted the activities of H_2_O_2_-detoxifying enzymes CAT and GPx in BJ cells following H_2_O_2_ exposure (500 µM; 1 h) in a concentration-dependent manner. DMSO (0.5%) was used as a vehicle control. Enzymatic activities were determined immediately after the treatments (*n* = 3; each biological replicate consisted of three independent wells; mean ± SEM). * *p* < 0.05 versus vehicle controls; ^†^ *p* < 0.05 versus H_2_O_2_-treated cells.

In addition to its direct neutralizing activity, the protective activities of eriodictyol against oxidative injury in dermal fibroblasts may be due to an increase in the activities of cellular antioxidant enzymes such as CAT and GPx. CAT is the main enzyme responsible for the detoxification of high H_2_O_2_ concentrations, whereas GPx is responsible for the elimination of low flux of H_2_O_2_ [37]. In this study, the activities of CAT and GPx in dermal fibroblasts were significantly decreased by 63% and 60%, respectively, following 1 h of H_2_O_2_ exposure (Figure 6A,B). Eriodictyol pretreatment enhanced the activities of both CAT and GPx in a concentration-dependent manner; specifically, 20 µM eriodictyol promoted the activities of CAT and GPx in BJ cells by 119% and 130%, respectively, compared with the results obtained when following H_2_O_2_ exposure alone (Figure 6A,B). These kinetic study results suggest that eriodictyol protects dermal fibroblasts from oxidative damage by upregulating the cellular capacity to eliminate oxidative stress.

### 3.7. Eriodictyol Inhibited H_2_O_2_-Induced Cytotoxicity by Induction of Cellular Antioxidant Enzyme Expression

**Figure 7:**
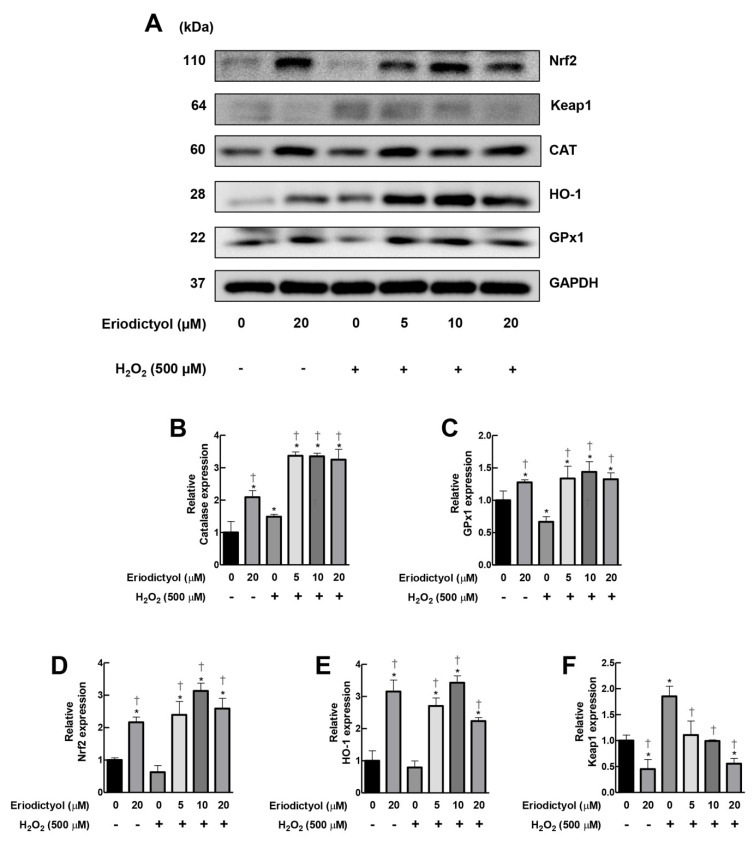
Eriodictyol promoted the expressions of CAT and GPx1 via Nrf2 cascade. (**A**) Western blot assay demonstrated that supplementation with eriodictyol (5–20 µM; 24 h) enhanced the expressions of CAT and GPx1 in BJ cells following H_2_O_2_ treatment (500 µM; 1 h). The upregulation of these H_2_O_2_-removal enzymes is possibly due to the activation of the Nrf2 pathway, as observed by an increase in the expression levels of Nrf-2 and its downstream target HO-1 and a decrease in Keap1 protein expression. GAPDH was used as a loading control. DMSO (0.5%) was used as a vehicle control. Results are representative of three independent experiments. Full unedited blots are shown in Supplementary Figure S2. (**B**–**F**). The band intensity of certain proteins from Western blot analysis was quantified with Image J software and normalized to loading control GAPDH. Data represent mean ± SEM of protein expression with respect to untreated control (*n* = 3). * *p* < 0.05 versus vehicle controls; ^†^ *p* < 0.05 versus H_2_O_2_-treated cells.

Another potential pharmacological activity for the protective effects of eriodictyol against oxidative damage is an upregulation of the expression of antioxidant machinery. A Western blot analysis demonstrated that eriodictyol (20 µM) promoted the expressions of CAT and GPX1 by 110% and 30%, respectively, compared to untreated BJ cells (Figure 7A–C). Moreover, supplementation with eriodictyol (5–20 µM) significantly upregulated both H_2_O_2_-removal enzymes in response to oxidative insult by H_2_O_2_ (Figure 7A–C). Hence, treatment with eriodictyol not only enhanced enzymatic activity (Figure 6) but also promoted the expression of the H_2_O_2_-removal system of dermal fibroblasts (Figure 7A–C).

The nuclear factor erythroid 2-related factor (Nrf2) pathway is a master regulator of redox homeostasis and antioxidative response [38]. The activation of this pathway leads to an upregulation of several types of cellular antioxidant machinery, including CAT and GPx1 [39], resulting in increased cellular resistance to oxidative insults. Hence, we postulated that the increase in the expressions of CAT and GPX1 due to eriodictyol could be regulated by the Nrf2 pathway. We demonstrated that the level of expression of total Nrf2 in BJ cells with eriodictyol (20 µM) was increased by 220% compared with the untreated control (Figure 7A,D). Pre-treatment with eriodictyol (5–20 µM) upregulated total Nrf2 protein levels following H_2_O_2_ exposure (Figure 7A,D). In addition to CAT and GPx1, the expression of another downstream Nrf2 target, heme oxygenase-1 (HO-1), was observed. HO-1 is an inducible antioxidant enzyme that plays a pivotal role in cellular protection to combat deleterious ROS from oxidative insults [40]. HO-1 expression was upregulated by 330% with eriodictyol treatment (20 µM) compared with non-treated fibroblasts. Eriodictyol (5–20 µM) significantly promoted the level of the HO-1 protein in response to H_2_O_2_ challenge (Figure 7A,D). These data suggest that the Nrf2 pathway could be a major contributor to the beneficial activities of eriodictyol.

Moreover, we also observed the effects of eriodictyol on the expression of Kelch-like ECH-associated protein 1 (Keap1), an inhibitory regulator of Nrf2. Under a physiological condition, Keap1 represses Nrf2 expression by targeting Nrf2 to ubiquitin-mediated degradation. In the presence of oxidative stress, Nrf2 is liberated from Keap1, enters into the nucleus, and induces the expressions of various sets of cytoprotective proteins, e.g., antioxidant, anti-inflammatory, and detoxification enzymes [41]. Parallel to Nrf2 protein levels, eriodictyol (20 µM) suppressed the expression of Keap1 compared with untreated BJ cells (Figure 7A,F). Exposure to H_2_O_2_ dramatically enhanced Keap1 protein levels by 85% (Figure 7A,F). Pre-treatment with eriodictyol significantly inhibited H_2_O_2_-induced-Keap1 upregulation in a dose-dependent fashion (Figure 7A,F). These results suggest that eriodictyol could activate the Nrf2 cascade of dermal fibroblasts by decreasing the expression of its negative regulator, Keap1. Altogether, the Western blot results demonstrate that the Nrf2 pathway could be a key regulator for the beneficial activities of eriodictyol against oxidative injury in dermal fibroblasts.

## 4. Discussion

This study provides novel and useful insights into the beneficial actions of eriodictyol against oxidative stress in dermal fibroblasts. The cell viability results show that eriodictyol significantly prevented H_2_O_2_-induced cytotoxicity in dermal fibroblasts (Figure 1C). The fluorescence microscopic study with Hoechst 33342/PI dual staining further demonstrated that necrotic cell death could be a principal contributor to H_2_O_2_–induced toxicity under this experimental condition (Figure 2). It is important to note that the characteristics of cell death from H_2_O_2_ treatment likely depend on concentration. A moderate concentration of H_2_O_2_ leads to the induction of apoptosis, while the elevated concentration of H_2_O_2_ triggers necrosis [42,43,44,45]. We observed that eriodictyol could prevent the harmful effects of H_2_O_2_ through the inhibition of necrosis (Figure 2). Our study indicates that the protective effects of eriodictyol on dermal fibroblasts (Figure 1C and Figure 2) are strongly associated with its antioxidant activities. We examined whether the protective effects of eriodictyol on dermal fibroblasts are due to oxidative-stress alleviation. The DCFH-DA assay results suggest that eriodictyol inhibits the H_2_O_2_-induced oxidative stress in a concentration-dependent manner (Figure 3B). However, there are several limitations regarding the use of DCFH-DA as an indicator to monitor the cellular redox status, especially its selectivity and sensitivity [26,46]. To overcome these potential drawbacks, we further confirmed the DCFH-DA assay results by measuring the intracellular GSH levels following treatment. Reduced glutathione is an important marker of cellular redox homeostasis. One of the major roles of GSH is ROS neutralization to prevent oxidative damage. Therefore, changes in intracellular GSH levels could reflect the oxidative status of cells [36]. Consistent with the DCFH-DA assay results, we found that eriodictyol preserved the availability of GSH in dermal fibroblasts following H_2_O_2_ exposure in a concentration-dependent manner (Figure 4). These results strengthen the hypothesis that the protective activities of eriodictyol on dermal fibroblasts are due to the inhibition of oxidative stress.

We propose three potential pharmacological activities underlying the protective effects of eriodictyol against oxidative imbalance in dermal fibroblasts (Graphical abstract). First, eriodictyol could directly neutralize intracellular ROS, as eriodictyol significantly inhibited DPPH free radical activity in a concentration-dependent manner (Figure 5). For these proposed activities, significant amounts of eriodictyol have to accumulate and be presented in fibroblasts to combat oxidative insults. Hence, additional studies on the cellular uptake profile of eriodictyol are necessary to support this mechanism. In addition to direct scavenging activities, eriodictyol could act as a metal-chelating agent. Due to its chemical structure, eriodictyol can coordinate endogenous redox-active metal and form a complex to prevent the initiation and/or progression of oxidative stress. Eriodictyol contains two possible chelating sites for metal ions, including the 4-carbonyl-5-hydroxyl site of A and C rings and the catechol moiety of a B ring (3′-4′ site) [47]. Further investigations are needed to explore the contribution of metal-chelating properties on the preventive effects of eriodictyol on fibroblasts.

Second, eriodictyol enhances the capacity of dermal fibroblasts to eliminate detrimental ROS. In this study, we demonstrated that eriodictyol could enhance CAT and GPx activities (Figure 6), resulting in the increased capability of fibroblasts to detoxify H_2_O_2_. Third, eriodictyol promotes the expression of CAT and GPx1 of dermal fibroblasts (Figure 7). The results of the Western blot analysis clearly show that the increase in the expression levels of H_2_O_2_-removal enzymes by eriodictyol was associated with the regulation of the Nrf2 cascade. Treatment with eriodictyol increased the Nrf2 protein levels of dermal fibroblasts, leading to an upregulation of Nrf2 downstream antioxidant targets, including HO-1, CAT, and GPx1, thereby enhancing the cellular antioxidant capacity. We also showed that stimulation of the Nrf2 pathway by eriodictyol is possibly due to a downregulation of Keap1 protein levels (Figure 7).

The results of our present study are consistent with previous findings on eriodictyol relative to different models of diseases. For in vitro models of oxidative-stress-related disorders, including cardiovascular disease [19], age-related macular degeneration [22], diabetic retinopathy [48], and neurodegenerative disease [49], eriodictyol inhibits oxidative injury through the upregulation of the Nrf2 network. Eriodictyol (10 µM) prevented the cytotoxicity of H_2_O_2_ (300 µM) in human umbilical vein endothelial cells through the induction of the ERK/Nrf2/ARE/HO-1 pathway [19]. In addition, eriodictyol (50 µM) protected human adult retinal epithelial cells from t-BOOH-induced-injury by the regulation of Nrf2 cascades [22]. Furthermore, treatment with eriodictyol (5–20 µM) inhibited the harmful effects of high glucose in rat retinal ganglial cells by the activation on the Nrf2 system and the enhancement of the enzymatic activity of cellular antioxidant systems (CAT, GPx, and superoxide dismutase (SOD)) [48]. Lastly, eriodictyol (20–80 µM) suppressed H_2_O_2_-induced-neurotoxicity (200 µM) in rat pheochromocytoma cells via the stimulation of the Nrf2/ARE signaling system [49]. It is important to note that the effective concentration of eriodictyol in this present study (5–20 µM) is closely comparable with previous reports using different in vitro models (5–80 µM). For in vivo studies, preclinical models of acute lung injury [20], kidney injury [50], and liver injury [51] demonstrated that eriodictyol possesses beneficial effects against oxidative damage that manifest via the regulation of the Nrf2 cascade. Eriodictyol exerted its protective activities on mice with LPS-induced-lung-injury by the activation of the Nrf2 system and the increased expression of redox regulatory protein thioredoxin-1 in lung tissue [20]. Additionally, supplementation with eriodictyol inhibited cisplatin-induced-nephrotoxicity in mice via the upregulation of Nrf2/HO-1 expression and the increased activities of antioxidant defensive enzymes (CAT, GPx, and SOD) [50]. The association between the pharmacological activities of eriodictyol and the enhancement of the capacities of antioxidant enzymes via the Nrf2 pathway was also reported in rats with arsenic-induced hepatotoxicity [51]. These previous studies support our present finding that the protective effects of eriodictyol in dermal fibroblasts are tightly regulated by the Nrf2 signaling system.

Fibroblasts play vital roles in maintaining the structural integrity and elasticity of skin tissue [52]. An excess of oxidative stress can cause a reduction in the proliferative ability and the functional impairment of fibroblasts, which eventually results in the appearance of aging features, such as wrinkles, dehydration, hyperpigmentation, and loss of skin elasticity [53]. Our study demonstrates that eriodictyol could increase the resistance of fibroblasts to oxidative injury by enhancing the capacity of cells to eliminate harmful oxidant H_2_O_2_. Hence, eriodictyol would preserve the youthful morphology and functional activity of fibroblasts, support cell proliferation, and delay the aging phenotypes.

## 5. Conclusions

Eriodictyol exerts protective actions against H_2_O_2_-induced cytotoxicity in dermal fibroblasts by inhibiting oxidative stress. The enhancement of the capacity of antioxidant machinery to detoxify ROS appears to be a principal factor for these beneficial activities of eriodictyol. Our findings suggest that supplementation with eriodictyol could effectively enhance skin viability, promote skin elasticity and increase skin resilience. Altogether, eriodictyol could be a promising candidate for anti-aging purposes in pharmaceutical and cosmeceutical products.

## Data Availability

Not applicable.

## Supplementary Materials

The following supporting information can be downloaded at: https://www.mdpi.com/article/10.3390/nu14122553/s1, Figure S1: Apoptotic and necrotic cells were not immediately observed after H_2_O_2_ exposure. BJ cells were incubated with eriodictyol (5–20 µM; 24 h), then exposed to H_2_O_2_ (500 µM, 1 h). The mode of cell death was instantly investigated after H_2_O_2_ challenge using Hoechst 33342/PI double-staining approach (n = 3; magnification = 20x; scale bar = 100 µm); Figure S2: Full unedited western blots for Nrf2 (110 kDa), Keap1 (64 kDa), CAT (60 kDa), HO-1 (28 kDa), GPx1 (22 kDa) and GAPDH (37 kDa). These western blot images represent three biological replicates and are used to prepare Figure 7.

## Abbreviations

ATCC, American Type Culture Collection; CAT, catalase; DCFH, 2′,7′-dichloro-dihydro-fluorescein; DMSO, dimethyl sulfoxide; DPPH, 2,2-diphenyl-1-picrylhydrazyl; GSH, glutathione; HO-1, heme oxygenase-1; MEM, minimum essential medium; LPS, lipopolysaccharide; MeOH, methyl alcohol; MTT, 3-(4,5-dimethylthiazol-2-yl)-2,5-diphenyltetrazolium bromide; Nrf2, nuclear factor erythroid 2-related factor; PBS, phosphate-buffered saline; PI, propidium iodide; ROS, reactive oxygen species; RSA, radical scavenging activity; SEM, standard errors of the mean; SOD, superoxide dismutase.
