# Supplementary material for: Eriodictyol Attenuates H2O2-Induced Oxidative Damage in Human Dermal Fibroblasts through Enhanced Capacity of Antioxidant Machinery
Source: Nutrients. 2022 Jun 20;14(12):2553. doi: 10.3390/nu14122553 (PMC9228723; doi:10.3390/nu14122553)
Supplement: Supplementary file 1 [file nutrients-14-02553-s001.zip › nutrients-1733667-supplementary.pdf]

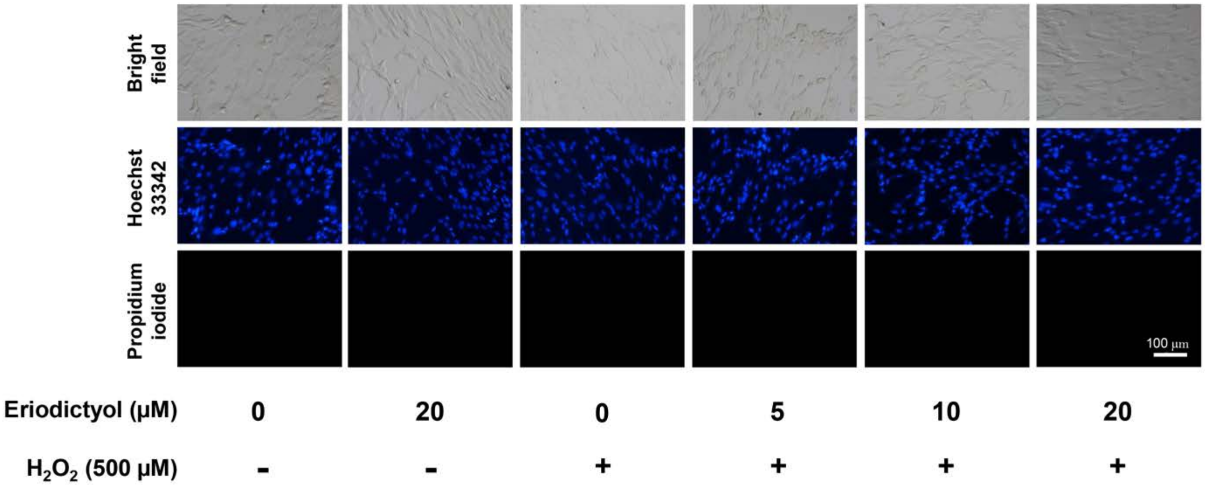

Figure S1. Apoptotic and necrotic cells were not immediately observed after H_2O_2 exposure. BJ cells were incubated with eriodictyol (5 - 20 μM; 24 h), then exposed to H_2O_2 (500 μM, 1 h). The mode of cell death was instantly investigated after H_2O_2 challenge using Hoechst 33342/PI double-staining approach (*n* =3; magnification = 20x; scale bar = 100 μm).

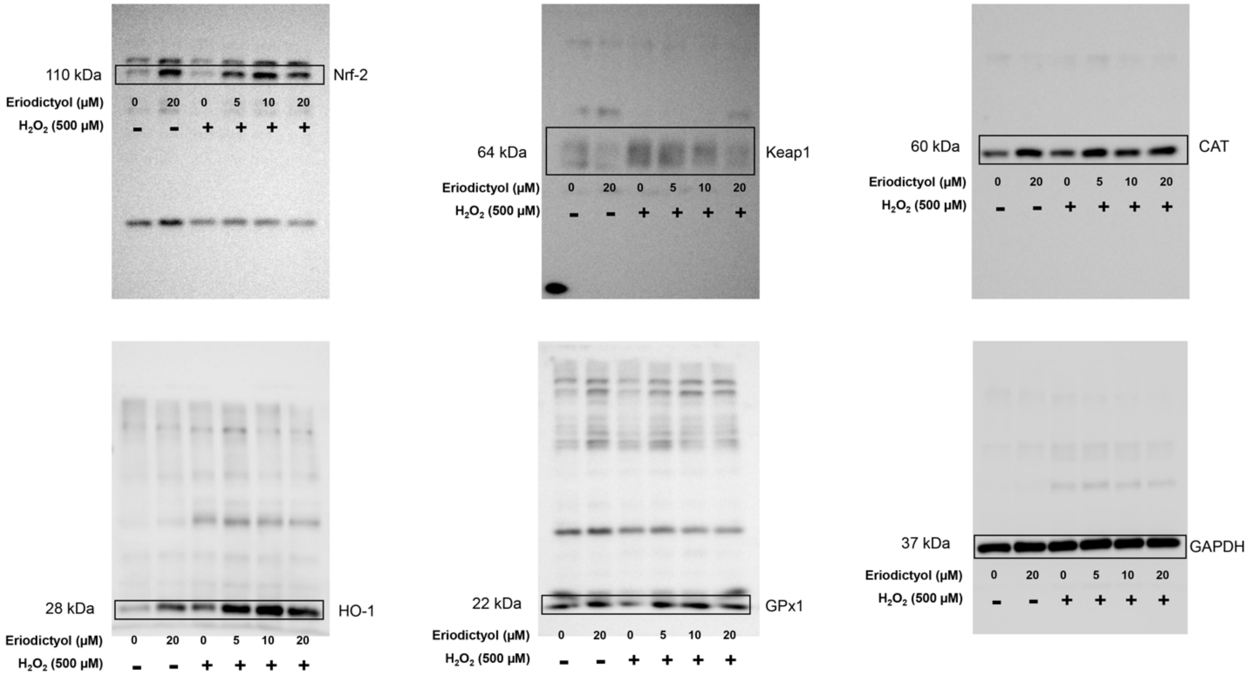

Figure S2. Full unedited western blots for Nrf2 (110 kDa), Keap1 (64 kDa), CAT (60 kDa), HO-1 (28 kDa), GPx1 (22 kDa) and GAPDH (37 kDa). These western blot images represent three biological replicates and are used to prepare Figure 7.
